# Drug Addiction in Gay and Bisexual Men Living with HIV Engaged in Sexualized Drug Use: Recent Drug Use, Polydrug and Depressive Symptoms as Predictors

**DOI:** 10.1007/s10461-025-04695-x

**Published:** 2025-04-05

**Authors:** Mar J. F. Ollero, Pablo Ryan, Helen Dolengevich-Segal, Joanna Cano-Smith, Luis Ramos-Ruperto, Alfonso Cabello, Matilde Sanchez-Conde, Noemí Cabello, Jose Sanz, Lucio Jesus García-Fraile, Leire Perez-Latorre, Otilia Bisbal, Sara De La Fuente, Juan Emilio Losa, Alicia González-Baeza

**Affiliations:** 1 Biological and Health Psychology Department, Universidad Autónoma de Madrid, Campus de Cantoblanco, C/Iván Paulov 6, Madrid, 28049 Spain; 2 Internal Medicine and HIV Unit, Infanta Leonor University Hospital, Madrid, 28031 Spain; 3 Department of Medicine, Medicine Faculty, Complutense University of Madrid (UCM), Madrid, 28040 Spain; 4 Biomedical Research Network Center in Infectious Diseases (CIBERINFEC), Instituto de Salud Carlos III, Madrid, 28034 Spain; 5 Dual Pathology Program, Psychiatry Service, Henares Hospital, Avda Marie Curie, Madrid, 28822 Coslada Spain; 6 Infectious Diseases and HIV Unit, La Paz University Hospital, IdiPAZ, Madrid, 28046 Spain; 7 Infectious Diseases and HIV Unit, Hospital Universitario Fundación Jimenez Diaz y IIS-FJD, Madrid, 28040 Spain; 8 Infectious Diseases and HIV Unit, Ramon y Cajal University Hospital, Madrid, 28034 Spain; 9 Internal Medicine Department, San Carlos University Hospital, Madrid, 28040 Spain; 10 Complutense University, Madrid, Spain; 11 CIBER de Enfermedades Infecciosas (CIBERINFEC), Carlos III Health Institute, Madrid, Spain; 12 Internal Medicine, Alcalá University Hospital, Madrid, 28805 Spain; 13 Internal Medicine and HIV Unit, La Princesa University Hospital, Madrid, 28006 Spain; 14 HIV Unit, Gregorio Marañon University Hospital and IiSGM, Madrid, 28009 Spain; 15 Internal Medicine and HIV Unit, 12 de Octubre University Hospital, Madrid, 28041 Spain; 16 Internal Medicine and HIV Unit, Puerta del Hierro University Hospital, Majadahonda, 28222 Spain; 17 Infectious Diseases, Fundación Alcorcón University Hospital, Alcorcón, 28922 Spain

**Keywords:** Drug-related problems, Drug use, Polydrug use, *Chemsex*, GBMSM, HIV

## Abstract

Evidence shows that engaging in sexualized drug use (SDU) can be associated with sexual health problems and poor mental health. However, the prevalence of drug-related problems associated with SDU remains unclear. Our study aimed to examine the prevalence and associated factors of drug-related problems and drug dependence in a sample of gay, bisexual, and other men who have sex with men living with HIV (HIV + GBMSM). We included 101 HIV + GBMSM who had engaged in SDU in the last year. Participants completed an online survey featuring a validated questionnaire (the DUDIT test) to assess the risk of drug-related problems and drug dependence. Univariate and multivariate analyses were conducted to explore variables associated with drug-related problems. 80% of our sample had symptoms suggestive of drug-related problems, with 5% showing likely drug dependence. Additionally, 10% had suffered an overdose with loss of consciousness, 9% experienced suicidal thoughts associated with SDU, and approximately 20% had sexual difficulties during sober sex since using drugs for sex. Multivariate analysis identified that recent drug use (less than 15 days prior), polydrug use, and depressive symptoms are independent predictors of drug-related problems. Our study revealed a high prevalence of drug-related problems among HIV + GBMSM engaged in SDU. The factors associated with drug-related problems identified in our study can serve as key markers in clinical settings where HIV + GBMSM receive care. These indicators can help detect community members most at risk and facilitate the provision of resources and interventions to prevent SDU-related harm.

## Resumen

La evidencia disponible muestra que el uso sexualizado de drogas (USD) se asocia a problemas de salud sexual y mental, pero se desconoce la prevalencia de problemas relacionados con las drogas en este contexto. Nuestro estudio tuvo como objetivo explorar la prevalencia de uso problemático de drogas y sus factores asociados en una muestra de hombres homosexuales, bisexuales y otros hombres que tienen sexo con hombres con el VIH (HSH VIH+). Un total de 101 HSH VIH + que habían participado en USD en el último año completaron una encuesta que incluyó un cuestionario validado (test DUDIT) para evaluar el uso problemático y dependencia a drogas. Se realizaron análisis univariantes y multivariantes. El 80% de nuestra muestra presentó indicadores de uso problemático de drogas y el 5% probable dependencia de drogas. Además, el 10% había sufrido sobredosis con pérdida de conciencia, el 9% pensamientos suicidas asociados con el USD y el 20% dificultades durante el sexo sin drogas. El uso reciente de drogas (menos de 15 días antes), el uso de múltiples drogas y los síntomas depresivos fueron predictores independientes de uso problemático de drogas. Nuestro estudio muestra una alta prevalencia de problemas relacionados con las drogas entre los HSH VIH + que habían realizado USD. Además, los factores asociados identificados podrían servir como indicadores de riesgo en recursos clínicos donde los HSH VIH + reciben atención, pudiendo ayudar a detectar a los miembros de la comunidad con mayor riesgo y facilitar la provisión de recursos e intervenciones para prevenir daños relacionados.

## Introduction

Sexualized Drug Use (SDU) refers to an expanding phenomenon where individuals engage in sexual intercourse while using drugs, either before or during the encounter, to enhance pleasure, extend the duration, and intensify the overall experience [1]. SDU sessions can last for several hours or even days and often involve multiple sexual partners [2].

Individuals engaged in SDU are predominantly gay men, bisexuals, and other men who have sex with men (GBMSM). It has been suggested that SDU is a form of drug use specific to this population [3]. Therefore, analyzing the sociocultural and psychological factors affecting GBMSM is essential for a more comprehensive understanding of the phenomenon [4]. In this regard, previous research has identified societal attitudes toward homosexuality, higher rates of trauma, and HIV/AIDS-related stigma as contributing factors to the prevalence of SDU among GBMSM [3].

The prevalence of SDU among GBMSM varies across studies, sub-population groups, and definitions of SDU. A recent meta-analysis estimated that 16% of GBMSM in Europe engage in *Chemsex* [5]. In Spain, the prevalence of SDU among the general GBMSM population ranges from 14.1% [6] to 21.1%, based on lifetime prevalence shown in the latest study [7]. Higher prevalence rates have been observed among GBMSM attending sexual health clinics [8], and in those GBMSM living with HIV (HIV + GBMSM), among whom prevalence rates range between 29% and 48% [1, 9–11].

Negative consequences on physical, mental, and sexual health have been described among GBMSM engaged in SDU. In a study by Platteau et al. [12], 40.2% of GBMSM engaged in SDU reported negative impacts on physical health, 64.6% on mental health, and 37.8% on sexual health. Physical health risks include severe drug intoxications, overdoses with loss of consciousness and comas, neurological and cardiovascular complications, viral and bacterial infections, and in extreme situations, death [13]. Regarding sexual health, high rates of sexually transmitted infections (e.g., HIV, syphilis, gonorrhea), lack of respect for sexual boundaries during SDU sessions, difficulty having sex without drugs, and loss of control over sexual life have been documented [14, 15].

Several studies have established a connection between SDU and mental health issues [16]. GBMSM engaged in SDU are at a higher risk of experiencing psychotic symptoms [17], suicidal ideation and attempts [18, 19], as well as depression or anxiety [20]. Specifically, GBMSM who engage in intravenous drug use (*slamsex*), inhaled methamphetamine, and use several drugs concurrently (polydrug use) face an increased risk of mental health issues [18]. Moreover, substances commonly used during SDU sessions, such as cathinones, methamphetamine, gamma-Hydroxybutyric acid (GHB), or ketamine, are recognized as potentially addictive drugs [21–24]. Similar to other addictive substances, the presence or absence of drug-related problems or symptoms of addiction distinguishes between recreational and problematic SDU [25].

The intersection between SDU and HIV is complex and continues to raise questions. Research suggests that engaging in SDU may increase the risk of HIV transmission through condomless sex or bloodborne exposure [18, 26]. Furthermore, HIV + GBMSM tend to report more frequent engagement in risky sexual behaviors and drug use than their HIV-negative counterparts, potentially leading to higher rates of SDU and drug-related problems, or worse consequences related to SDU [27]. Moreover, a recent review indicates that HIV + GBMSM engaged in SDU may face specific risks, such as loss of adherence to antiretroviral therapy, missed medical appointments, and impaired immunological functioning due to drug use. Additionally, pharmacological interactions between some antiretrovirals and recreational drugs may increase the risk of drug intoxication or overdose [28]. To date, the prevalence and types of drug-related problems in GBMSM engaged in SDU remain unknown, with only two studies reporting high rates of drug-related issues in specific sub-groups of HIV-positive and HIV-negative GBMSM [29, 30].

The present analysis aimed to examine the prevalence and types of drug-related problems among HIV + GBMSM engaged in SDU. Additionally, we aimed to compare participants with and without drug-related problems to identify associated factors that could serve as potential indicators for detecting these problems.

## Methods

### Procedures

The present analysis is a sub-analysis conducted within the U-SEX 2 GESIDA 9416 study, which took place in 20 HIV clinics across Madrid from October 2019 to February 2020 [31]. The USEX-2 study was designed to calculate the prevalence of SDU in a sample of HIV + GBMSM and explore associated factors, comparing participants who engaged in SDU in the past year (*n* = 317) to those who did not (*n* = 107). HIV + GBMSM, attending one of the 22 regional HIV clinics involved in the study, and aged 18 or older, were recruited to participate in an online survey. The researchers of the study provided each participant with a card during their routine medical visit. Each card contained a unique code to ensure that only those with a card could complete the survey. The study was prematurely interrupted due to the COVID-19 pandemic, with a total of 424 participants. The comprehensive methodology of the study has been published elsewhere [31].

This sub-analysis includes only the group of HIV + GBMSM engaged in SDU, aiming to examine the prevalence and types of drug-related problems, and their associated factors in this sub-sample. Participants who did not fully complete the Drug Use Disorders Identification Test (DUDIT) for identifying drug-related problems were excluded.

Sexualized drug use (SDU) was defined as the intentional consumption of mephedrone or other cathinones, 3,4-methylenedioxy-N-methylamphetamine (MDMA), methamphetamine, amphetamines, GHB/GBL, ketamine, or cocaine during sexual activity over the past year.

This research adhered to the ethical standards of the Declaration of Helsinki and received approval from the Ethics Committee of Hospital Universitario Gregorio Marañon (HUIL 1606-13/2019). Data collection and management were executed using the Research Electronic Data Capture (REDCap) system, hosted by ‘Asociación Ideas for Health,’ a Spanish non-profit dedicated to research and medical education [32].

### Measurements and Instruments

The survey included self-reported questions covering various domains: sociodemographic data, HIV infection status, sexual behaviors, sexually transmitted infections (STIs), mental health history, substance use history and SDU related questions. Additionally, we incorporated validated instruments to evaluate drug-related problems, and symptoms of anxiety and depression.

A total of 105 questions were created ad hoc to evaluate the previously mentioned domains. Regarding variables associated with SDU, we asked about the time since consumption began, places of consumption, average session duration, monthly money expenditure on drugs, and negative consequences associated with SDU. Specifically, to assess the negative consequences three ad hoc questions were created. First, we asked about sexual consequences since using drugs during sex, where participants reported about whether or not they had experienced each of the following: “I have enjoyed drug-free sex less than before,” “I have had erection problems or premature or delayed ejaculation when I have had drug-free sex,” “I have stopped having drug-free sex,” and “none of these have happened to me in the last year.” Secondly, we asked about negative consequences induced by drugs, with response options including: “adverse physical effects while under the influence of the drug (e.g., tachycardia, nausea, vomiting),” “any overdose with loss of consciousness,” “anxiety attacks or panic attacks,” “irritability or aggressiveness,” “paranoia (ideas of being persecuted or that they wanted to harm me),” “suicidal thoughts,” “suicide attempts,” and “none of these.” Third, we asked whether drug use had caused any problems in the areas of work, social life, family life, or control of HIV infection. We considered participants to have daily interference if they reported problems in any of these areas.

The survey also included the following self-reported, validated questionnaires:

The Drug Use Disorders Identification test (DUDIT; [33]). This instrument screens for drug-related problems using 11 items that explore various aspects of drug use and dependence. Responses are rated on a 0 to 4 point Likert scale, with a maximum score of 44. Higher scores suggest a greater likelihood of drug-related problems. Scores of 6 or more indicate harmful drug habits or probable drug-related problems, while scores above 24 suggest probable heavy drug dependence.

The Hospital Anxiety and Depression Scale (HADS; [34]; Spanish version [35]),. This 14-item scale includes two 7-item subscales to measure symptoms of anxiety and depression. Responses are rated on a 0 to 3 point Likert scale, with a total maximum score of 42 and a subscale maximum of 21. Participants with scores of 8 or higher on either subscale are considered to have clinically significant symptoms of depression or anxiety, respectively [36].

### Statistical Analysis

To characterize the sample and the drugs used by participants, we calculated absolute and relative frequencies for categorical variables and mean (standard deviation) for continuous variables. We had a maximum of one missing value per variable, with percentages calculated over the number of total cases (*N* = 101).

Comparative analyses between participants with and without indicative scores of drug-related problems were conducted using univariate binary logistic regression. The variables included in the univariate logistic regressions can be found in Table 2. Some variables presented estimation problems, as they either did not produce estimates or had abnormally wide confidence intervals. Descriptive statistics are therefore presented for these variables, but they were excluded from the analysis due to an unequal distribution of frequencies (i.e., a cell with zero or one observation in the contingency table). These variables were: unprotected anal intercourse, sex work, *slamsex*, SDU sessions longer than 12 h, monthly drug expenditure above 100 euros, daily interference caused by SDU, cessation of drug-free sex, suicidal thoughts, overdose with loss of consciousness, paranoia, anxiety/panic attacks, and irritability or aggressiveness.

Next, a stepwise multivariate binary logistic regression model was conducted to determine which variables consistently suggested drug-related problems when all variables were considered concurrently. The multivariate analysis only included independent variables that were statistically significant in the univariate analysis (*p* <.05). Drug-related problems was the dependent variable in all analyses.

Data analysis was performed using R Studio, version 2023.03.0 [37]. Results were considered statistically significant at a p-value of less than 0.05.

## Results

Of the total 424 HIV + GBMSM who completed valid surveys in the USEX-2 Study, this analysis included those who had engaged in SDU over the previous year (*n* = 107). Six participants were excluded due to incomplete DUDIT test (i.e., any unanswered item), which made it impossible to determine if they had drug-related problems. Thus, our final sample included 101 HIV + GBMSM who were engaged in SDU.

### Sociodemographic, Medical Characteristics and Patterns of Drug Consumption in the Entire Sample

Participants were mostly Spanish born (*n* = 71; 70.30%), with a significant proportion from South or Central America (*n* = 25; 24.75%). The participants were predominantly middle-aged (Mean = 38.44; SD = 8.07), with a smaller percentage being over 50 years old (*n* = 9; 8.91%). Educational levels were high; 35.64% (*n* = 36) had completed secondary education, and 58.42% (*n* = 59) held a university degree. Employment was common among the participants; 74.25% (*n* = 75) were actively employed, 74.25% (*n* = 75) earned over 1,000 euros monthly, and 25.74% (*n* = 26) earned more than 2,000 euros monthly. The average salary in Spain in 2020 was €2.097,13 per month, and the minimum interprofessional salary for that year was set at €950 per month [38]. Therefore, all participants had earnings above the minimum interprofessional salary.

Regarding relationship status, 36.63% (*n* = 37) had a stable partner, of whom 51.35% (*n* = 19) stated that their relationship had lasted at least 2 years.

All participants were sexually active, with 67.33% (*n* = 68) reporting more than 24 sexual partners annually. A significant majority (94.96%, *n* = 95) reported unprotected anal intercourse (UAI) in the past year. Additionally, 43.56% (*n* = 44) practiced fisting, and 7.92% (*n* = 8) were involved in male sex work.

Regarding HIV-related variables and other sexually transmitted infections (STIs), participants had been living with an HIV diagnosis for an average of 7.89 years (SD = 5.84). All the participants were on antiretroviral treatment (ART) when they completed the survey, with 96.05% (*n* = 94) reporting an adherence of over 90% and 63.37% (*n* = 64) achieving complete adherence. Moreover, 63.37% (*n* = 64) had been diagnosed with another STI in the previous six months.

Table 1 shows the pattern of SDU among participants. Mephedrone and GHB were the most used drugs, each consumed by 70.30% (*n* = 71) of the sample, followed by cocaine, methamphetamine, ketamine and MDMA. Additionally, a high rate (*n* = 84, 83.2%) reported frequent use of alkyl nitrites (poppers). Predominant modes of drug administration included snorting mephedrone and cocaine, taking GHB and MDMA orally, and inhaling methamphetamine. The table also notes a relatively frequent practice of *slamsex* (*n* = 17, 16.83%), with mephedrone and methamphetamine being the most injected substances. Anal use of mephedrone, methamphetamine, cocaine and ketamine were also reported. Moreover, more than half of the participants (*n* = 56, 55.44%) reported polydrug use, defined as combining three or more drugs during sessions, and a significant number of participants (*n* = 78, 77.22%) reported drug use in the last month. Lastly, nearly a quarter of participants typically engaged in sessions exceeding 12 h and spent over 100 euros on drugs monthly (Table 1).

**Table 1:** Pattern of drug use and SDU-related variables in the entire sample of participants (*n* = 101)

Variable	*n* (%)
***Type of drug and route of administration (N = 101)***	
*Mephedrone*	71 (70.30)
Snorted	67 (94.37)
Anal	18 (25.35)
Injected	14 (19.72)
Oral	7 (9.86)
Smoked	6 (8.45)
*Cocaine*	44 (43.56)
Snorted	43 (97.73)
Smoked	7 (15.90)
Anal	4 (9.09)
Oral	4 (9.09)
Injected	3 (6.81)
*Methamphetamine*	36 (35.64)
Inhaled	35 (97.22)
Injected	10 (27.78)
Snorted	4 (11.11)
Anal	4 (11.11)
Oral	1 (2.78)
*Ketamine*	29 (28.71)
Snorted	27 (93.10)
Anal	6 (20.70)
Injected	3 (10.34)
Oral	2 (6.89)
*GHB (oral)*	71 (70.30)
*Akilyl nitrites / poppers (inhaled)*	84 (83.17)
*Ecstasy (oral)*	27 (26.73)
***Pattern of drug use and SDU-related variables (N = 101)***	
*Drug last consumed*	
< 15 days	59 (58.41)
15 days–1 month	19 (18.81)
> 1 month	23 (22.77)
*Slamsex*	17 (16.83)
*Polydrug use*	56 (55.45)
*Sessions > 12 h*	25 (24.75)
*Monthly drug spending > € 100*	29 (28.71)

Type of drug and route of administration

### Drug-related Problems, Anxiety and Depressive Symptoms, and Consequences of SDU

In our sample of 101 HIV + GBMSM engaged in SDU, 80.20% (*n* = 81) had scores in the DUDIT test indicative of drug-related problems (score ≥ 6). Of all, 4.95% (*n* = 5) were likely to have drug dependence (DUDIT test score ≥ 25). Additionally, 39.60% (*n* = 40) of participants had scores suggesting clinically relevant anxiety symptoms (HADS-A ≥ 8), and 18.81% (*n* = 19) significant depressive symptoms (HADS-D ≥ 8).

**Fig. 1:**
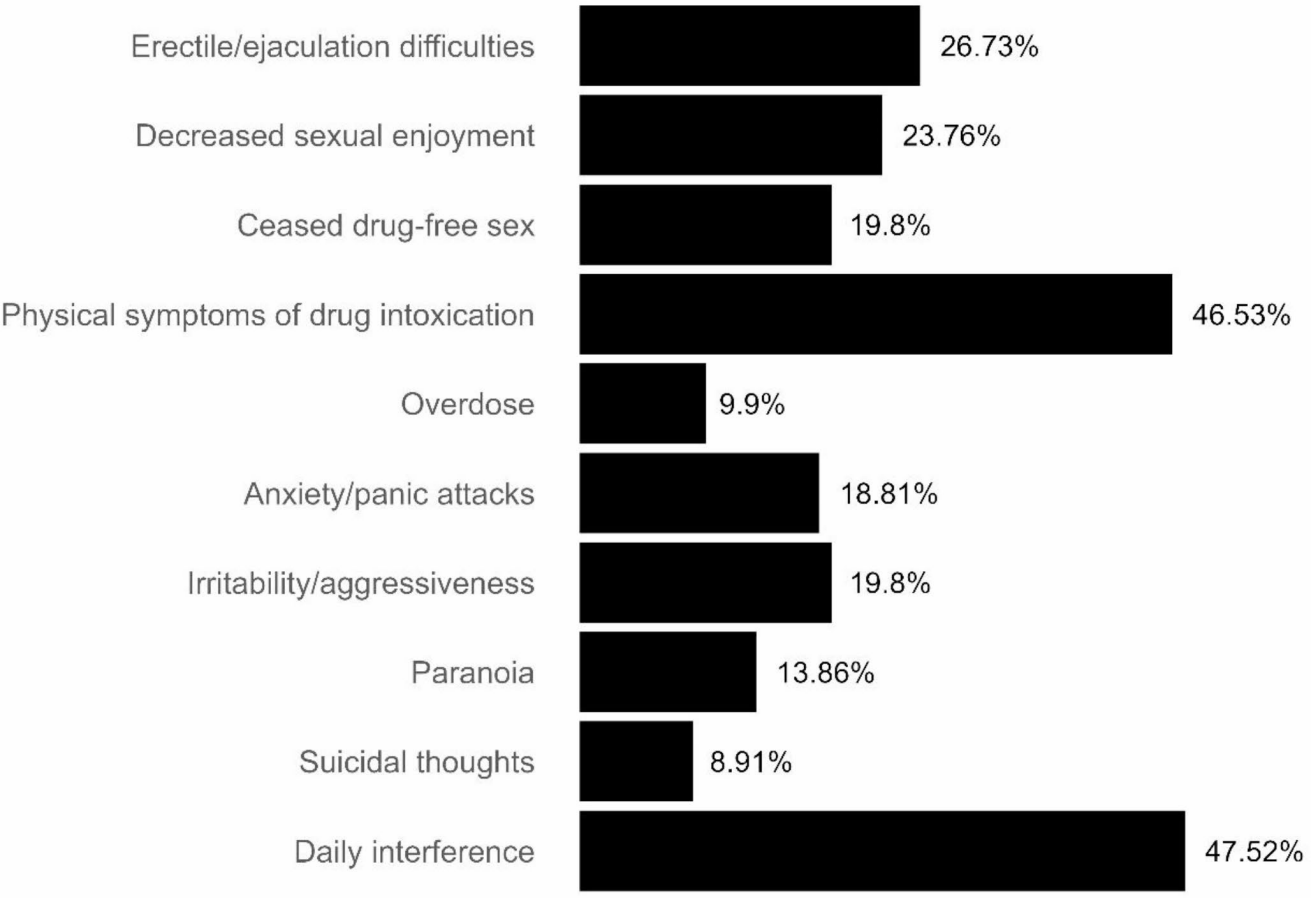
Consequences associated to Sexualized Drug Use in our sample (*n* = 101)

Figure 1 shows the negative consequences of SDU as reported by the study participants. Approximately a quarter of the participants reported erectile difficulties, as well as premature or delayed ejaculation. Close to 24% (*n* = 24) noted a decrease in sexual enjoyment during drug-free sex and 20% (*n* = 20) ceased engaging in sober sex after initiating SDU. Nearly half reported physical symptoms of drug intoxication, such as tachycardia, nausea, and vomiting. Other effects induced by drugs were reported in 10–20% of cases: overdoses with loss of consciousness, anxiety/panic attacks, irritability or aggressive episodes, paranoia or suicidal thoughts related to SDU. Finally, nearly half experienced interference with their daily activities caused by SDU.

### Comparison Between Participants with and without Drug-Related Problems: Predictors of Problematic Drug Use

Our univariate analysis (Table 2) revealed that drug-related problems were associated with the absence of a romantic partner, the use of inhaled methamphetamine and GHB, higher levels of anxiety and depressive symptoms, drug use within 15 days prior to the survey, polydrug use during sessions and, experiencing adverse physical effects while under the influence of drugs.

In the multivariate analysis, the stepwise regression method indicated that the model with the best fit (Nagelkerke R²=0.51) included the following variables: being single (OR (CI) = 2.89 (0.79–11.48), *p* =..11), last drug use less than 15 days ago (OR (CI) = 8.37 (2.21–39.52), *p* <.01), polydrug use during sessions (OR (CI) = 10.84 (2.92–51.70), *p* <.01), and depressive symptoms (OR (CI) = 1.24 (1.01–1.65), *p* =.07). A third model, excluding the non-significant variable “being single,” was fitted, as shown in Table 2. The adjustment index showed minimal change compared to the previous model (Nagelkerke R^2^=0.48). This model reported independent significant associations between drug-related problems and recent drug use (less than 15 days ago), polydrug use during sessions, and higher depressive symptoms.

**Table 2:** Univariate and Stepwise multivariate logistic regression with problematic drug use as a dependent variable (*n* = 101)

Univariate				Multivariate (*N* = 97)	
Variable	*N*	OR (CI)	*p*-value	OR (CI)	*p*-value
Spanish born	100	0.60 (0.16–1.84)	0.40		
Age	101	0.98 (0.92–1.04)	0.47		
High Education	101	1.25 (0.19–24.7)	0.84		
Employed	100	2.15 (0.64–9.84)	0.26		
Salary > 1000	101	0.95 (0.28–2.81)	0.93		
**Single**	**100**	**3.30 (1.22–9.40)**	** 0.02**		
Relationship > 2 years	37	0.39 (0.09–1.59)	0.20		
Sexual patners > 24	100	2.03 (0.73–5.56)	0.17		
Fisting	100	1.23 (0.46–3.44)	0.69		
Years HIV	101	0.99 (0.91–1.08)	0.82		
Perfect adherence ART	101	0.92 (0.31–2.50)	0.87		
STI (last 6 months)	101	0.51 (0.15–1.46)	0.23		
Mefedrone	101	1.36 (0.46–3.77)	0.56		
Cocaine	101	2.79 (0.98–9.22)	0.07		
**Metamphetamine**	**101**	**3.90 (1.19–17.64)**	** 0.04**		
Ketamine	101	4.50 (1.18–29.62)	0.05		
**GHB**	**101**	**3.05 (1.10–8.51)**	** 0.03**		
Popper	101	0.84 (0.18–2.96)	0.81		
Extasis	101	1.59 (0.52–5.98)	0.45		
**Drug last consummed < 15 days**	**101**	**8.46 (2.79–31.85)**	**> 0.01**	**8.32 (2.29–36.96)**	**< 0.01**
**Polydrug use**	**100**	**7.43 (2.45–27.90)**	**> 0.01**	**10.20 (2.86–45.66)**	**< 0.01**
**Anxiety**	**100**	**1.22 (1.06–1.43)**	** 0.01**		
**Depression**	**100**	**1.37 (1.11–1.84)**	** 0.01**	**1.28 (1.03–1.71)**	**< 0.05**
Ejaculation	101	1.59 (0.52–5.98)	0.45		
Less enjoyment	101	3.36 (0.87–22.20)	0.12		
**Adverse physical effects**	**101**	**3.23 (1.13–10.70)**	** 0.04**		

Variables in bold indicate a p-value significance of *p* ≤ 0.05

## Discussion

The present analysis provides novel findings regarding drug-related problems associated with SDU in a sample of HIV + GBMSM. Notably, 80% of the HIV + GBMSM engaged in SDU had indicators of drug-related problems, with 5% potentially having drug dependence. A relatively high proportion of participants experienced negative consequences associated with SDU, including nearly half who suffered physical symptoms of drug intoxication, 10% having suffered an overdose with loss of consciousness, and 9% experiencing suicidal thoughts related to SDU. Additionally, approximately 20–25% reported sexual difficulties during sober sex since engaging in SDU, such as diminished sexual enjoyment, erection or ejaculation difficulties, or ceasing to engage in sex without drugs. Finally, almost half of the participants reported that SDU had interfered with work, social, family, or control of HIV infection.

To our knowledge, few studies have systematically evaluated the prevalence of drug-related problems in individuals engaged in SDU. Peyriere et al. [30] used the Substance Involvement Screening Test (ASSIST) questionnaire to assess these issues in a sample of men who have sex with men receiving HIV pre-exposure prophylaxis (PrEP) who were engaged in *Chemsex*. They found that 67.8% of the sample indicated drug-related problems requiring brief intervention, and 4.4% exhibited signs of drug addiction or dependence. In another study we reported, the DUDIT test indicated a higher rate of drug dependence (34%) among HIV + and HIV-negative GBMSM engaged in SDU. However, these rates are not directly comparable to those in the current analysis, as all participants were involved in a community program designed to support individuals engaged in SDU [29]. Additionally, Torres et al. [39] reported lower rates (48%) of moderate to high risk of drug-related problems in a sample of men who have sex with men in Brazil using the ASSIST questionnaire. The variance in drugs included in the definition of SDU renders the samples non-comparable. In our study, a significant majority (86.1%) reported using traditional *Chemsex*-related substances such as cathinones, crystal methamphetamine, or GHB during sexual activity, while the remainder (13.9%) used cocaine, MDMA, amphetamines, or ketamine. In contrast, Torres et al. [39] included a broader range of illicit drugs, including cannabis, cocaine/crack, hallucinogens, or inhalants during sex. The high rate of drug-related problems in our sample aligns with the increased risk of frequent drug use and polydrug use previously reported in HIV + GBMSM compared with HIV-negative GBMSM [27, 40]. Furthermore, the similar rates found in our study and by Peyriere et al. [30] suggest that GBMSM in PrEP programs and those living with HIV may share a comparably high risk of experiencing drug-related problems. Therefore, higher rates of recreational drug use might predominate in other GBMSM sub-groups engaged in SDU with lower rates of drug-related problems.

In addition to drug-related problems, negative consequences induced by drugs, such as symptoms of intoxication and overdose, have been frequently reported among individuals engaged in SDU [41, 42]. While these consequences associated with SDU have been described, prevalence data are limited. Thus, we have previously found a prevalence of around 15% of paranoid ideation and drug-induced loss of consciousness in another sample of HIV + GBMSM engaged in SDU [18]. The same study reported that 15.3% and 13.8% of the participants, respectively, experienced suicidal ideation and suicide attempts associated with drug use [18]. More recently, Blanc et al. [43] found that 24% of GBMSM with problematic *Chemsex* reported having had at least one suicide attempt. This frequency is higher than what we found as they reported lifetime suicide attempts, whereas our study reported drug-induced suicidal ideation and attempt. Previous studies have found a higher risk of suicidal ideation in men engaged in SDU compared to those who were not [44]. Several factors may contribute to this higher prevalence, especially in HIV + GBMSM. A recent systematic review reported that synthetic cathinones, such as mephedrone or 4-MMC, were associated with self-harm and suicide attempts, particularly when combined with other drugs [45]. *Slamsex* has also been associated with higher rates of suicidal ideation or attempts while under the effects of drugs [18]. In addition to the effects induced by the drugs themselves, other risk factors can predispose individuals to suicide in the general population. Some of these factors include psychiatric disorders, physical illness, unemployment, lower educational level, previous contact with the justice system, or parental death by suicide [46]. Particularly in the LGTBIQ + population, adverse childhood experiences, internalized queerphobia, minority stress, interpersonal violence, bullying, familial conflict, anti-LGBTQ + policies [47] or having experienced non-consensual sex [48] have been proposed as risk factors for suicidality. Finally, among HIV + GBMSM, suicide attempts have been associated with HIV stigma, with higher risks for those who were socially excluded, rejected by a sexual partner, verbally abused, or physically abused for living with HIV [49]. Other risk factors found among people living with HIV include alcohol and cocaine dependence, major depressive disorder, and post-traumatic stress disorder [50].

Sexual health consequences associated with SDU have also been reported in several studies [1, 16, 51, 52]. However, the impact of SDU on an individual’s sexuality remains unclear. A recent study revealed that 16.7% of GBMSM engaged in SDU ceased to have sober sex, and 57.4% sometimes felt a loss of control over their sexual life [15]. These results are consistent with our study, which reveals that 19.8% of men ceased to have sober sex after starting SDU. The effects of SDU on sober sex and overall sexuality warrant thorough investigation in GBMSM engaged in SDU, as this could help to identify SDU-related problems beyond or with drug-related issues. We believe that recovery programs should include strategies aimed at restoring pleasure in sober sex and promoting healthy sexuality.

In addition to the previously discussed results, our study has identified for the first time factors associated with drug-related problems in GBMSM engaged in SDU. Participants who engage in polydrug use during SDU were ten times more likely to experience drug-related problems, while those who had used drugs in the 15 days prior to completing the survey (recently SDU) were eight times more likely. Moreover, an increase in depressive symptoms was associated with a higher likelihood of drug-related problems. Although polydrug use has not been directly linked to drug-related problems in people engaged in SDU, it has previously been associated with severe consequences, such as induced psychotic symptoms, regardless of *slamsex* [18], and with a higher risk of STIs [53]. Polydrug use has also been identified as a predictor of drug-related problems in other populations [54, 55]. Furthermore, the association with recent drug use could be identifying cases or more frequent and active drug use. Finally, while the association between depressive symptoms and SDU have been previously described [20], the association with drug-related problems has been unexplored in people engaged in SDU. This finding is aligned with previous studies in other populations, showing that psychoactive drugs and their withdrawal syndrome can induce depressive symptoms [56, 57], and that individuals with depression are at a higher risk of developing substance use disorders [58, 59]. Compared to the general population, rates of depression are higher among both GBMSM and people living with HIV. Internalized lesbian, gay, bisexual, transgender, queer (LGBTQ+) prejudice, stress from hiding one’s sexual identity, and maladaptive coping strategies are some of the risk factors for depression identified for the LGBTQ + population [60]. Furthermore, HIV-related stigma, virological effects on the central nervous system, and neurotoxicity associated with antiretroviral therapy are risk factors for depression among HIV-positive individuals [60, 61].

In our opinion, this study provides novel findings and a valuable basis from which to propose future studies. However, certain limitations must be acknowledged, particularly regarding our sample. The small sample size, although common in studies focusing in GBMSM engaged in SDU due to the specificity of this population, may affect the robustness of the results and the statistical power of the analyses. Furthermore, since all participants are from the same city and country, the sample may exhibit high homogeneity, potentially limiting its representativeness. This raises the possibility for unaccounted masked variables and restricts the generalizability of the results to other groups of HIV + GBMSM engaged in SDU. Additionally, due to the small sample size, we found a class imbalance (i.e. 81 drug-related problems versus 20 non-drug-related problems). Class imbalance in drug-related problems is to be expected in SDU since the drugs used have a high addictive potential but a larger sample size could mitigate the effect of the imbalance. This class imbalance contributes to bias in the results and reduces precision and statistical power of our analysis. As noted in the Methods section, it led to some estimation problems that prevented us from extracting conclusions from some of the variables collected. This study also has limitations related to its design. The cross-sectional approach does not allow for establishing causal relationships between variables. Additionally, the use of self-reported survey data introduces the risk of recall bias, which was mitigated by limiting the timeframe for memory-dependent questions.

Studying drug-related problems in GBMSM engaged in SDU presents significant challenges, and this study represents a valuable step forward. To further investigate this phenomenon, future research aiming to determine the prevalence and characterization of drug-related problems should compare HIV-positive and HIV-negative GBMSM engaged in SDU, exploring whether there is an increased risk and specific factors that make HIV + GBMSM more vulnerable to experiencing drug-related problems. Our results also suggest that the prevalence and severity of consequences induced by drugs should be explored in depth, particularly addressing the interaction between different variables that may predispose HIV + GBMSM engaged in SDU to suicidal ideation and attempts or to experiencing physical symptoms of drug intoxication. Moreover, further studies are needed to explore the factors and their interaction that predispose some HIV + GBMSM engaged in SDU to experience depressive symptoms, which in our study is a factor associated with drug-related problems. All these new studies would lead to a better understanding of the phenomenon, early detection of people at risk, and allow for the design of more effective interventions for HIV + GBMSM engaged in SDU. Regarding other studies, prevalence and characterization studies of drug-related problems in different subgroups of GBMSM engaged in SDU could also determine whether other groups are particularly vulnerable. Furthermore, researchers interested in studying this phenomenon in depth should avoid class imbalance by collecting larger samples and conducting power studies to ensure the reliability of their results. Besides that, they could consider including more heterogeneous samples and longitudinal designs.

Despite its limitations, our study has significant strengths. The comprehensive evaluation of participants across sociodemographic, medical, and behavioral domains, coupled with the use of standardized questionnaires to assess drug-related problems, anxiety, and depression, reinforce the robustness of our results. Moreover, the main demographic and behavioral characteristics of our sample are aligned with those reported in other European and Spanish studies on GBMSM engaged in SDU [15, 62–64]. This includes a predominance of Spanish-born men, as well as a notable proportion from Central and South America, primarily of middle age, with advanced education, earning moderate to high monthly incomes, with well-controlled HIV, high levels of adherence to antiretroviral therapy, and a high frequency of sexual risk behaviors. Our participants also reflect other cohorts of HIV + GBMSM engaged in SDU in terms of drug use patterns, with a high frequency of mephedrone (70%) and GHB/GBL use (70%), and significant percentages of cocaine (43%), methamphetamine (35%), and MDMA (27%) use. *Slamsex* was reported by 17% of the sample, involving the injection of mephedrone, methamphetamine, cocaine, and ketamine. Additionally, inhaled methamphetamine (35%), anal drug use, and polydrug use (55%) were common. Similar drug use patterns have been previously reported in England [2] and Asia [50], though cocaine use during sex appears to be more common in Spain than in other countries [11, 15].

Finally, we believe that our results and those yielded by other future studies could be particularly useful in clinical settings such as HIV Units or sexual health clinics where HIV + GBMSM are treated. From our results, health professionals who care for HIV + GBMSM can be aware that a large proportion of those engaged in SDU may present drug-related problems and negative associated consequences that affect different areas of their quality of life. This should encourage them to carry out evaluation protocols to detect these problems and develop preventive interventions to avoid the development of substance use disorders. Furthermore, the associated factors to drug-related problems we identified might help these professionals in detecting cases with higher risk when patients report recent SDU, polydrug use during sessions and/or depressive symptoms. It can be particularly useful in hospital settings and community centers where professionals serve many people with little time for each person or where professionals are not addiction specialists. The presence of these signs may highlight the need for more in-depth evaluations or referrals to other specialists.
